# Supporting quality public and patient engagement in health system organizations: development and usability testing of the Public and Patient Engagement Evaluation Tool

**DOI:** 10.1111/hex.12378

**Published:** 2015-06-25

**Authors:** Julia Abelson, Kathy Li, Geoff Wilson, Kristin Shields, Colleen Schneider, Sarah Boesveld

**Affiliations:** ^1^ McMaster UniversityHamiltonONCanada; ^2^ Ontario Ministry of Health and Long‐Term CareTorontoONCanada; ^3^ Nova Scotia Health AuthorityHalifaxNSCanada; ^4^ Allied Health and Outreach ServicesNorWest Community Health CentresThunder BayONCanada; ^5^ Winnipeg Regional Health AuthorityWinnipegMBCanada

**Keywords:** public and patient engagement in health system decision making, public and patient engagement evaluation, public and patient involvement

## Abstract

**Objectives:**

Only rudimentary tools exist to support health system organizations to evaluate their public and patient engagement (PPE) activities. This study responds to this gap by developing a generic evaluation tool for use in a wide range of organizations.

**Methods:**

The evaluation tool was developed through an iterative, collaborative process informed by a review of published and grey literature and with the input of Canadian PPE researchers and practitioners. Over a 3‐year period, structured e‐mail, telephone and face‐to‐face exchanges, including a modified Delphi process, were used to produce an evaluation tool that includes core principles of high‐quality engagement, expected outcomes for each principle and three unique evaluation questionnaires that were tested and revised with input from 65 end users.

**Results:**

The tool is structured around four core principles of ‘quality engagement’: (i) integrity of design and process; (ii) influence and impact; (iii) participatory culture; and (iv) collaboration and common purpose. Three unique questionnaires were developed to assess each of these four evaluation domains from the following perspectives: (i) those who participate in PPE activities; (ii) those who plan, execute or sponsor PPE activities within organizations; and (iii) those who provide the leadership and capacity for PPE within their organizations.

**Conclusions:**

This is the first known collaboration of researchers and practitioners in the co‐design of a comprehensive PPE evaluation tool aimed at three distinct respondent groups and for use in a wide range of health system organization settings.

## Introduction

The practice of involving citizens and patients in health‐system planning, priority setting and policy making has evolved considerably over the last decade. Efforts to consult, involve and engage citizens and patients are supported by a growing array of frameworks and models, and organizations have become more sophisticated at matching engagement methods to their goals, decision‐making contexts and affected populations.^1^, ^2^, ^3^, ^4^, ^5^ As public and patient engagement (PPE) – an inclusive term used to capture a wide range of efforts aimed at actively involving citizens and patients in various domains and stages of health system decision making – becomes more embedded in health organizations, a call to rigourously evaluate, and establish an evidence base for these activities, has followed suit. The dearth of evidence to support the growing field of PPE has been a recurring theme in the PPE literature.^6^, ^7^, ^8^, ^9^, ^10^ Few comprehensive frameworks exist for assessing the quality and impacts of PPE in health system decision making. However, the UK patient and public involvement (PPI) in health research literature has recently contributed important foundational work on impact assessment,^11^, ^12^, ^13^, ^14^ and outside the health field, several notable evaluation frameworks exist.^15^, ^16^, ^17^, ^18^, ^19^, ^20^ Still, efforts to produce practical, easy‐to‐administer evaluation tools for use in a broad array of health system organizations are best described as nascent, most have been tailored to specific organizational, decision‐making or population contexts, limiting their application to broader settings.^21^, ^22^, ^23^

This study takes an important first step towards addressing the PPE evaluation gap by sharing the results of a 3‐year collaboration of Canadian researchers and practitioners to develop a PPE evaluation tool for use across a wide range of health system organizations. The tool aims to lay the foundation for creating a robust base of evidence that can inform and improve practice in this area.

### Study context and background

New legislative mandates for PPE, and its evaluation were introduced in several Canadian provinces in the mid‐2000s.^24^, ^25^, ^26^ These replaced or expanded on existing PPE efforts in many provinces, contributing to a growing proliferation of strategies, frameworks and toolkits in this area.^27^, ^28^, ^29^ Recognizing the importance of generating a knowledge base to support this growing field, a pan‐Canadian partnership of PPE practitioners and researchers formed with the shared goal of developing a *common evaluation tool* that could be used in a variety of health system organizations and that would collectively contribute to improvements in the quality of PPE practice across the country. The partnership grew out of, and was supported by, two successive research grants from the Canadian Institutes of Health Research (including contributions from health system practice partners), all focused on the evaluation of PPE in health system decision making. Referred to as the *research–practice collaborative*, the partnership included representation from seven provinces, six regional health authorities and two provincial and local health organizations.

## Methods

The evaluation tool was developed through an iterative, collaborative and consensus‐based process designed to ensure relevance, acceptability and uptake in a wide range of organizations. The tool was developed through the following three discrete phases: (i) a review of published and grey literature on PPE evaluation; (ii) structured e‐mail, phone and face‐to‐face exchanges with the research–practice collaborative to (a) agree on the core principles of quality PPE that would guide the development of the tool; (b) map the core principles to outcomes and prioritize these for inclusion in the tool; and (c) develop three discrete evaluation questionnaires for three different respondent groups; and (iii) testing of the usability of the questionnaires preceding final revisions to the tool.

### Phase 1: Review of evaluation literature and resources

The published and grey literature was reviewed with a focus on evaluation tools and frameworks designed for use in broad health system or public policy decision‐making processes. Relevant literature was identified through the systematic searching of English language published and grey literature covering the 1996–2009 time period, undertaken in conjunction with previous research studies led by the first author.^30^ These results were augmented by targeted searching of the health and social sciences literature between 2010 and 2014 using the key words ‘public OR citizen OR patient’ AND ‘engagement OR involvement OR participation’ AND ‘evaluation’. In addition to these resources, we reviewed the internal evaluation documents of practice partner organizations.

### Phase 2: Tool development process

#### (a) Developing the principles of ‘quality’ PPE

Members of the collaborative contributed at several stages to the identification of a set of overarching principles for carrying out high‐quality PPE activities that would serve as the foundation for the evaluation tool. University‐based members of the team prepared, and shared with practice partners, a document summarizing key findings from the published PPE evaluation literature and organizational documents reviewed in Phase 1. Principles were generated by the full team membership through an iterative process of identifying common ground across values statements, principles and recommendations extracted from literature and organizational documents. Each principle's relevance to the mission of the organizations represented through the collaborative was also reviewed to ensure that the tool was built on a common foundation across a wide range organizations operating in different political and organizational contexts.

#### (b) Moving from principles to outcome measures

The next step in the tool development process involved mapping the core principles to outcome measures that would serve as the starting point for the creation of the evaluation instrument. This pivotal work was carried out through a 2‐day workshop convened in February 2012, which included full representation from the research team and organizational partners across the seven participating provinces (*N* = 10). A professional facilitator was hired to manage the deliberations, and a background document was pre‐circulated to participants to guide meeting discussions. The main task for participants was to identify and agree on a core set of outcomes that could serve as the basis for the  development of the evaluation tool. The group was organized into break‐out groups throughout the meeting to facilitate comprehensive discussion. Reporting back and large group discussion ensured that all perspectives were carefully considered and for validation purposes.

Following the 2‐day workshop, outcomes were prioritized through a modified Delphi process administered through a series of telephone and e‐mail exchanges over 3 months. Practice partners engaged in an iterative process of group discussion and independent online rankings of provisional outcomes and indicators for each of the core principles identified under a). Areas of disagreement were resolved by consensus, and a final set of outcomes and indicators was agreed to that would be used as the basis for questionnaire development.

Construction of the draft evaluation instrument was guided by the outcome of the Delphi process and informed by a bank of sample questions compiled from evaluation resources identified in Phase 1. Relevant questions were used and/or adapted as necessary with consideration given to their ability to measure the outcomes comprehensibility (e.g., are the questions easy to understand), ease of administration (e.g., a moderate number of questions) and clarity of key terms (e.g., diversity of people and demographics vs. diversity of perspectives).

The results of this final prioritization and questionnaire development process were reviewed at a second face‐to‐face meeting of the collaborative in May 2013. Final revisions were made to the outcomes and draft evaluation questionnaires, and plans for the final stage of questionnaire development were agreed to.

#### (c) Developing and tailoring the evaluation instruments for different users

The mapping and prioritization of outcome measures formed the basis for the construction of the evaluation questionnaires that would be tailored to three discrete respondent groups: (i) participants in *ad hoc* or on‐going PPE activities; (ii) PPE project managers or directors working within organizations; and (iii) organizational leaders (e.g., senior management team and board members).

### Phase 3: Usability testing and revisions

A final stage of the tool development process involved testing the usability of the questionnaires with various respondent and end user groups. Practice partner members of the collaborative administered questionnaires to representatives from each of the respondent groups (i.e. participants, project managers and senior organizational personnel) in two health regions. Participants were asked to provide feedback on the structure, layout, comprehensibility, ease of use and utility of the questionnaire to relevant end users, individually through closed‐ and open‐ended questions at the end of the questionnaire (organization 1), and through requests for open‐ended e‐mail feedback (organization 2) (see Table 1).

**Table 1:** Description of usability testing phase

Participants	Feedback solicited
Citizens, patients and families/community advisory group members	Was the questionnaire easy to use?
Health authority program staff responsible for the planning and execution of PPE activities	Was the purpose of the questionnaire clearly stated?
Board members, senior management team, PPE directors and advisors	Were the instructions clear and helpful?
	Was the layout easy to follow?
	Were the questions easy to understand?
	Were there important questions missing?
	Do you think this questionnaire will be useful for our organization?
	How long did it take you to complete the questionnaire?
	Identify one way in which this questionnaire could be improved

## Results

### Literature review findings

The literature review yielded a small but influential set of documents that provided the foundation for identifying the core principles for quality PPE that would guide the development of our evaluation tool. For example, in their foundational work, inspired by Webler's meta‐principles of evaluation – fairness and competence^31^ – Rowe & Frewer^15^ identify two broad criteria which can be used to assess the effectiveness of public involvement processes: *acceptance criteria* which relate to the effective construction and implementation of a public involvement process and *process criteria* which relate to the eventual public acceptance of the outcome of the process. Acceptance criteria include a number of well‐known features of quality engagement such as representativeness and independence of participants, transparency of the process to the public and the influence of the process on the decision outcomes. Process criteria, as the label suggests, pertain to the features of the engagement process that ensure its credibility and legitimacy such as participants’ access to the resources required to fulfil their role, a clearly delineated task for participants and well‐documented ground rules for the participatory process. These principles were reinforced in subsequent reviews of the evaluation literature and proposed evaluation frameworks, which have documented increasing convergence around a core set of elements that define ‘quality’ PPE.^17^, ^19^

Our review of the PPE evaluation literature identified three notable gaps. First, most evaluation frameworks have been developed outside of the health field and, thus, may ignore important aspects related to evaluating the effectiveness of PPE in the health‐care context. For example, most health‐care organizations work closely with community partner agencies and consider engagement with these agencies central to their mandate. As a result, the inclusion of principles and outcomes measures that permit the assessment of the degree to which these collaborative relationships have been successful are important features of evaluation tools used in the health‐care domain.^22^ A second gap noted was, with few exceptions,^21^, ^22^ the lack of involvement of relevant stakeholders in the development of evaluation tools. Those studies that did involve end users noted the added value of involving these groups. A third gap identified was the absence of a segmented approach to PPE evaluation in terms of tailoring to different respondent groups and end users. Most evaluation frameworks are primarily focused on gathering participant or user feedback with little attention paid to the assessment of PPE initiatives from the project sponsor, internal PPE management or organizational leadership perspective. Practice partners viewed this gap as the most significant weakness in the PPE field and a major motivator for their interest in developing a more comprehensive tool that could meet multiple evaluation needs within their organizations.

Organizational and grey literature documents were also reviewed to assess the Canadian PPE practice community's current evaluation practice. Two health organizations were identified that had reasonably sophisticated strategies with considerable attention paid to evaluating different types and levels of engagement activity.^28^, ^29^ However, most were found to take a fairly rudimentary approach with a focus on assessing the procedural aspects of engagement processes using surveys of moderate quality. Minimal attention was given to assessing the impacts of these processes on individual participants, organizational decision making or community partners. Several other engagement frameworks and principles documents, drawn mostly from outside the health arena, provided additional guidance to inform the selection of core dimensions for a common evaluation tool.^7^, ^17^, ^18^, ^19^, ^20^, ^21^, ^27^, ^32^

### Results of tool development process

Through an iterative process of reviewing, comparing and reflecting on the different sources of evaluation material, the collaborative identified common ground on five principles that reflect the core dimensions of ‘quality PPE’ (Table 2). Subsequent discussions led to the merging of the first two principles to reflect the inter‐connectedness between PPE design and process features, which yielded four domains that would provide the organizing framework for the evaluation tool: (i) integrity of design and process; (ii) influence and impact; (iii) participatory culture; and (iv) collaboration and common purpose (Table 2).

**Table 2:** Principles of quality public and patient engagement and organizing framework for evaluation tool

Guiding principles for quality public and patient engagement (PPE)	Organizing framework
The principles of inclusivity, diversity, capacity and accessibility guide the selection, support and involvement of participants in all PPE activities	*Integrity of design and process* Assessing the integrity of the PPE design and implementation is central to ensuring that the process adheres to the features of high‐quality PPE
The principles of integrity, accountability and transparency guide the design and implementation of all PPE activities	The principles of inclusivity, diversity, capacity, accessibility, legitimacy, accountability and transparency guide the recruitment, selection, support and involvement of participants in all PPE activities
PPE activities are undertaken to influence and exert impacts on participants, organizations and decision making	*Influence and impact*
	The output obtained from high‐quality PPE activities is linked to relevant decision‐making processes within the organization
PPE activities are supported by a participatory culture	*Participatory culture*
	High‐quality PPE is supported by a culture of participation within the organization
PPE activities seek to promote the principles of collaboration, shared purpose and improved governance	*Collaboration and common purpose*
	High‐quality PPE supports and encourages internal and external stakeholders to work together to advance common goals

For each of these domains, a set of measurable outcomes was generated, also informed by the literature. These were then mapped to corresponding indicators, data collection sources and sample questions collected from partner organizations and the literature reviewed (Appendix S1). The final list of measures agreed to through the prioritization process is included in Table 3. These measures were then used as the basis for the development and tailoring of three unique evaluation questionnaires for the following respondent groups: (i) citizen and patient participants in engagement activities; (ii) managers and sponsors of PPE activities; and (iii) organizational leaders responsible for guiding and supporting PPE within their organizations.

**Table 3:** Final list of principles and prioritized outcomes

PPE Principle	Prioritized outcomes
Integrity of design and process	PPE participants represent the diverse range of views of those most affected by the decision
	Participants are provided with access to supports to enable participation, for example:
	PPE meeting‐related expenses are covered
	PPE activity location/amenities/time of day/day of event provide a comfortable, non‐threatening environment
	Relevant information, produced at an appropriate education level, is shared with participants
	Clear, two‐way communication exists between organizers and participants:
	The process and objectives are clearly communicated
	Participants understand how their input will be used
	Input from all participants is gathered through the process
	The outputs of the PPE activity process are reported to participants, including how their input will be used in the decision
Influence and impact	PPE informs planning/decision making
	PPE improves participant knowledge of:
	PPE issue
	Organization
	Health system
	Public/patient perspectives (if tailored to staff)
	Other topic of focus
	PPE leads to increased confidence/trust in:
	Providers
	PPE staff
	Organization as a whole
	Health system
	Personal competency (e.g., in diabetes management)
Participatory culture	The organization promotes and supports ongoing quality public engagement in strategic planning, policy and service delivery by:
	Embedding PPE values and principles in the organization's philosophy and structure
	Organizational leaders and managers have received training in PPE
	PPE practice is being implemented in service and policy work
	PPE is part of standardized business and planning processes
Collaboration and common purpose	The organization and other external community partners plan and work together to address the concerns of the people they serve

Each of the four evaluation domains is featured more or less prominently in the questionnaires to reflect their relevance and feasibility in relation to respondent groups. For example, elements in the integrity of design and process domain form the bulk of the participant questionnaire, as only perceptions of influence and impact can realistically be assessed in this respondent group. In contrast, the influence and impact domain features more prominently in the manager/sponsor and organization questionnaires given the interest of these end users in assessing the value added of engagement activities undertaken within the organization. Similarly, the participatory culture dimension is heavily emphasized in the organization questionnaire, less so in the manager/sponsor questionnaire and not at all in the participant questionnaire.

### Usability testing and revisions to the evaluation instruments

Practice partners in two health regions and provinces carried out the usability testing of the evaluation questionnaires in their respective organizations (organizations 1 and 2). The participant questionnaire was distributed to 145 public participants in total including members of community advisory councils, patients, family members and citizens who had participated in various engagement activities; 23 responses were received. The PPE manager/sponsor questionnaire was distributed to 28 directors and managers across both organizations with 14 responses received. The organizational leadership questionnaire was distributed to 75 health board member and senior management team members and directors across the two organizations with 28 responses received.

Usability testing of the *participant questionnaire* generated positive results. In organization 1, 85–100% of the total respondents (*N* = 13) indicated that the questionnaire was easy to use, had a clear purpose, helpful instructions, and was easy to understand and useful to the organization. Six respondents completed the open‐ended section with suggestions for improvement that included better linking of the questionnaire to specific engagement activities, improved wording clarity related to ‘being heard’ vs. ‘being listened to’ and additional room in the questionnaire for open‐ended comments. In organization 2, feedback obtained from the open‐ended e‐mail responses (*N* = 10) was also positive with general comments provided such as ‘survey questions are straightforward’, ‘questionnaire looks fine to me’, ‘it is very easy to understand’, ‘content is understandable’, ‘I found the questionnaire wording to be quite good’ and ‘it's precise, simple and to the point’. Areas for improvement included reducing the ambiguity of selected statements (e.g., ‘views of the most affected by the issue’, clarifying the distinction between confidence and trust), ensuring that the engagement activity that they are being asked to evaluate, and its objectives, are clearly described at the beginning of the questionnaire, the addition of an open‐ended question about how the participant sees the results of their involvement reflected in the organization's decisions and minor changes to layout to reduce confusion.

Usability testing of the *project questionnaire* generated more critical results with only 60–70% of respondents (*N* = 10) (organization 1) responding affirmatively in each of the domains. Nine respondents identified areas for improvement that included suggestions to use plainer and more concise language in the introduction to the questionnaire and its questions, the inclusion of more questions to assess the degree to which the organization engaged and with what impact, and improved layout of the questionnaire (e.g., larger font, more spacing). Feedback obtained from organization 2 focused on improving the wording of specific questions and providing greater clarity about the collaboration domain (e.g., examples of the different types of collaborators).

The *organization questionnaire* generated the most detailed and substantive feedback. Although respondents in organization 1 res‐ponded positively to the questionnaire (77–90% of respondents responding affirmatively in each of the domains of ease of use and understanding, layout, clarity of purpose, etc.), board members struggled with the questionnaire more than senior leadership team members. They asked for ‘less jargon’ and ‘more examples of engagement activities’ to orientate the respondent at the beginning of the questionnaire. One respondent stated that it was ‘too detailed for a volunteer Board member’ and more applicable to staff in the organization. Discussion with the team members in this organization explained that these findings were likely due to the recent appointment of a number of Board members who had not been fully oriented to the organization and its key areas of activity. A key area for improvement identified in both organizations was the need for greater specificity about the level of the organization addressed in some questions (e.g., programme area vs. organization as a whole).

Team members collectively reviewed the usability results, and a final round of revisions were made to the questionnaires to respond to these results. Final versions of each of the evaluation questionnaires are available at www.fhs.mcmaster.ca/publicandpatientengagement.

## Discussion

This pan‐Canadian research–practice collaborative has produced three unique evaluation questionnaires tailored to the following respondent groups: (i) public and patient participants; (ii) PPE sponsors and project managers; and (iii) organization leaders. To our knowledge, this is the first comprehensive effort to involve researchers and engagement practitioners in the design and usability testing of such a comprehensive evaluation tool. Our focus on developing short, easy‐to‐administer questionnaires, informed by evidence and practice, will facilitate its use in a wide range of organizations while also advancing the PPE evaluation field.

The collaborative process used to develop the evaluation tool models the principles of quality engagement itself, through the deliberate and extensive involvement of end users in each stage of the research process to ensure a practical, relevant product that reflects end user needs. Participating practice partners have already begun to use the evaluation tool and have included results from its early use in annual reports.^33^ One practice partner disseminated the organization questionnaire to 725 executives, managers and directors and received 307 completed questionnaires, which were summarized and distributed throughout the organization to summarize current knowledge about patient and public engagement practice.^34^

There are several weaknesses of the tool development process and its products that warrant attention and further refinement. First, while seeking to strike a balance between rigour and relevance, we are aware that the emphasis placed on user needs throughout the tool development process may have yielded a less robust evaluation tool with respect to its psychometric properties. For example, the focus on short, easy‐to‐administer questionnaires – for the participant respondent group in particular – may have compromised the tool's validity (e.g., number and specificity of statements used to assess a particular domain of practice, use of a 5‐point vs. a 7‐point scale). Further testing of each of the questionnaires is needed to determine and address any such weaknesses. Second, while patient and citizen perspectives on the core features of high‐quality engagement were captured in our literature review and, as a result, informed the tool development process, patients and members of the public were not directly involved in the tool development process beyond the usability‐testing phase. This may have led to the omission of dimensions that were important to this constituency. As we gain experience with the application of this tool, we also expect to gain a deeper understanding of how it is viewed by patients and the public, which will contribute to its amelioration over time. Third, the feasibility of applying the tool to every type, level and degree of patient and public engagement also needs further assessment. For example, some specificity may have been lost that may be important to particular settings or populations and that would necessitate additional tailoring. In addition, the adaptability of the participant questionnaire to evaluate both single event and on‐going engagement activities was questioned during the usability‐testing phase. Finally, the tool's focus on PPE evaluation in Canada's regionalized health‐care context may limit its applicability to non‐Canadian settings and to health‐care organizations that focus on smaller and more specialized populations. While plausible due to our reliance on Canadian sources of practice experience, this weakness is likely balanced by our extensive review of the international literature, which also informed our work, and by the large and highly diverse populations covered by the participating partner organizations (e.g., major urban centres, regional referral centres).

Given the shortfalls identified above, we view the evaluation tool presented here as an early step along a path of continuous improvement and refinement. It reflects the balance struck between the application of rigorous methods and relevance to practitioner needs. Given the considerable investments being made in PPE in health organizations around the world, this early step is critical to ensuring that this rapidly developing field is supported by a strong foundation of evidence.

## Conclusion

To our knowledge, this is the first collaboration of researchers and practitioners in the co‐design of a comprehensive evaluation tool aimed at assessing the quality and impact of episodic and on‐going PPE activities in health system organizations from three distinct perspectives – public and patient participants, sponsors and managers of PPE projects and organizational leaders responsible for PPE. We encourage further applications of the tool in other jurisdictions and organizational settings, and welcome their results to inform further refinement of the tool, with the collective aim of improving the practice and advancing the science of PPE.

## Funding sources

Canadian Institutes of Health Research (grant# MHS‐124759 and PHE‐91565).

## Conflicts of interest

None declared.

## Supporting information


**Appendix S1**: Preliminary mapping of PPE principles, measurable outcomes and data collection methods.
The evaluation questionnaires are available at: www.fhs.mcmaster.ca/publicandpatientengagement.
