# Supplementary material for: Supporting quality public and patient engagement in health system organizations: development and usability testing of the Public and Patient Engagement Evaluation Tool
Source: Health Expect. 2015 Jun 25;19(4):817–27. doi: 10.1111/hex.12378 (PMC5152717; doi:10.1111/hex.12378)
Supplement: Supplementary file 1 — Appendix S1: Preliminary mapping of PPE principles, measurable outcomes and data collection methods. The evaluation questionnaires are available at: www.fhs.mcmaster.ca/publicandpatientengagement. [file HEX-19-817-s001.pdf]

Appendix 1: Preliminary mapping of PPE principles, measurable outcomes and data collection methods

PPE Principle	Relevant PPE Outcomes	Indicator	Measurement tool / source of evidence		Sample questions drawn from evaluation documents collected
			Self-report	*Directly observed*	
**Integrity of design & process**	Our PE activities incorporate the core elements of quality PE:<br>• clearly communicated objectives<br>• independent, impartial facilitation<br>• provision of relevant background information<br>• responses to additional information needs<br>• structured discussion<br>• opportunity for a full range of views to be heard<br>• reporting back to participants	Sample indicators (there are many!):<br>• % reporting that objectives of the PE process were clear<br>• % reporting that the sponsoring organization explained how the input from the PE process was *intended* to be used<br>• % reporting that the meeting was managed in a neutral way (e.g. facilitation, rights to speak, etc.)<br>• % reporting that relevant information was provided to inform discussion<br>• % reporting that participants were able to adequately share their views<br>• documentation of the organization's use of PE input was communicated to all participants	Participant evaluations	Organizational documents (e.g., planning & recruitment materials, facilitator notes, presentation slides)<br><br>Qualitative analysis of PPE activity records can assess these elements more objectively	Was the mandate of the panel clearly explained? Were the objectives of the meeting clearly explained?<br>Did you receive enough information on the scope and purpose of this advisory committee? Did you understand your role and responsibilities on this committee?<br>Did you understand what aspects of this work you can influence?<br>Was the meeting managed in a neutral way (e.g. encouraged all to speak)? ; Are you able to express your views freely? Do you feel that your opinion matters and is understood?<br>Did participation allow the public to give adequate feedback on the analyses, alternatives and decisions about a policy or public action? Did the public have the opportunity to develop alternatives?<br>Did you feel you were able to contribute meaningfully to the meeting discussion?<br>Is there sufficient time at meetings to understand and discuss the issues?<br>Do you see how your committee's involvement has made a contribution to the work of the specific department? Did the research team clearly explain how the results of the meeting discussions will be used?
	Participants are provided with adequate support to contribute (e.g., information, respect, financial, etc.)	• % indicating that they had enough information to participate fully<br>• % reporting that they were listened to in a respectful manner<br>• % reporting that their expenses were covered at a reasonable level (determine a standard for this)	Participant evaluations		
	The opportunity for a diverse range of perspectives to be represented was provided	• % reporting that the quality of the invitation/recruitment materials encouraged a diverse range of perspectives to be represented<br>• % reporting that the PE activity encouraged a diverse range of perspectives to be represented	Staff evaluations<br><br>Self-evaluation of recruitment efforts as an indirect measure of inclusivity/diversity	Planning, recruitment and invitation materials	What steps did you employ in your process to reach out to groups who are typically under-represented or marginalized in public decision-making processes?

	A diverse range of perspectives was captured	• % of participants who felt that participants in PE activity were representative of relevant communities<br>• extent to which participants reflect the characteristics of the relevant population	Staff evaluations<br><br>Self-evaluation of inclusivity/diversity against a previously stated goal	Objective evaluation of diversity against population demographics	Who is participating in this PE process? To what extent are we recruiting the previously determined mix of people for the issue being discussed?<br><br>To what extent was the group reflective of the diversity of the community areas compared to census data?
**Influence & impact**<br>- tailor to: PE participants, Organization staff, Leadership, Community partners, PE practitioners	PE informs decision making and/or planning	• PE input was presented as part of decision making/planning about…<br>• PE input was discussed in meetings related to decision making/planning about…<br>• PE was referred to in documents related to decision making/planning about…	Participant, Council member surveys<br><br>Staff/senior management surveys, interviews	Review of organizational documents	What are public perceptions of the influence of involvement activities on decision-making and priority-setting?<br>Has the involvement of public members on this committee contributed to your department's work? If yes, please list some specific examples. If no or unsure, please tell us why<br>Has there been any new information for you about community needs or perspectives as a result of having public members on this advisory committee? If yes, please list some specific examples. If no or unsure, please tell us why<br>Do CE Officers respond to and appropriately refer the information needs of council in a timely manner<br>To what extent were the CHAC reports valuable in providing the Board with community perspectives about the issues? To what extent did the CHAC reports provide information to assist in decision-making related to those issues explored by the Council?
	PE leads to improved knowledge (knowledge-related outcomes could be tailored to PE issue, organization, health system, etc.)	• % of participants acquiring new knowledge through the PE initiative(s)	Repeated measures participant surveys to assess short- medium- and long-term effects; separate questions would need to be developed for each outcome of interest (e.g., attitudes, knowledge, etc.)	Review of organizational documents	Are participants better informed about the issue(s) that were addressed in the participatory program?<br>Did participation help participants cultivate skills such as eloquence, rhetorical ability, courtesy, imagination, and reasoning capacity?<br>Did participation help people clarify, understand, and refine their own preferences and positions on the issue(s)?<br>Did participation change participants' views on the issue(s)? Did participation help people take more account of community or collective concerns?<br>Did participation increase the likelihood that individuals will participate in future activities?<br>Were there any changes to the participants' perceptions, attitudes, knowledge, competence, skills, capacities or actions?<br>Did participation build community capacity to address current and future issues? Did participation identify and address community

					concerns, needs, and interests?
	PE produces increased confidence in:<br>• individuals (e.g., providers, themselves)<br>• organization(s) (to be specified)<br>• local/provincial health system	• % reporting increased confidence in…	Baseline and follow-up surveys (PE participants only or PE participants + public opinion)	Review of organizational documents	Were there any changes to the participants' confidence and willingness to get involved in the future?<br>Did participation increase participants' perceptions of political efficacy, sophistication, interest, trust, respect, empathy, and public-spiritedness?
	PE leads to increased trust in:<br>• individuals (e.g., providers)<br>• organization(s) (to be specified)<br>• local/provincial health system PE informs decision making/planning	• % reporting increased trust in…	Baseline and follow-up surveys (PE participants only or PE participants + public opinion survey)	Review of organizational documents	
**Participatory culture**	Quality PE is supported throughout the organization	• % of staff provided with opportunities to obtain PE expertise<br>• % of directors recommending PE training for their staff teams	Staff, senior management surveys, interviews<br>Participants, council member surveys/interviews<br>Organization-wide PE/CE survey	Budget commitments for PE staffing, training, programming & evaluation (on-going review)	What internal impacts and changes (i.e. structural changes, policy compliance, training, leadership development, attitudes/practices of staff and managers) have resulted from engagement<br>Are service structures compatible with community participation?<br>What is the overall attitude towards and understanding of community engagement with the organization?<br>Is there regular integration of engagement data as decision-making evidence? Note: could be directly measured once concept of "integration" is defined and operationalized<br>Has the involvement of public members on this committee contributed to your department's work? Why or why not?<br>Has the involvement of public members on this committee affected your department's opinion of patient & public involvement? Why or why not?<br>Are there any tools, methods or aspects of patient/public involvement that you would use in the future as part of your work?<br>Is there commitment from AHS leaders to support the work of councils?

**Collaboration & common purpose**	PE activities provide mechanisms for community partners to work together<br><br>PE activities support the identification of shared goals<br><br>PE activities support the achievement of shared goals	• % of community partner agencies involved in joint initiatives supported by the organization<br>• % of community partner agencies reporting that PE assisted in identifying shared goals<br>• % of community partner agencies reporting that PE assisted in the achievement of shared goals	Surveys and interviews with relevant stakeholders<br><br>Council member evaluations		Did it build trust and collaborative relationships with stakeholder groups? Did it increase consensus? Did it reduce conflict? Did it affect polarization? (Natabachi, 2012)<br>Do CEO officers provide adequate support to allow citizens to fulfill duties as a council member?
